# Supporting and understanding non-binary & gender diverse youth: a physician’s view

**DOI:** 10.1186/s13034-024-00798-w

**Published:** 2024-08-24

**Authors:** Jamie Agapoff

**Affiliations:** Department of Psychiatry, University of Hawai‘i at Mānoa, John A. Burns School of Medicine, Honolulu, Hawai‘i USA

Non-binary is a gender identity term describing a person whose gender exists between or outside the gender binary. Non-binary is also an umbrella term encompassing other gender identities such as genderqueer, genderfluid, agender, demigirl, demiboy, bigender, and others. As non-binary persons identify with a gender other than the one assigned at birth, they may be classified as transgender, although some may not identify with that label [1].

Population estimates of transgender and gender diverse (TGD) youth are varied [2–4]. Among youth ages 13 to 17 in the U.S., about 1.4% (~ 300,000) identify as transgender [3], and 1 in 4 identify as non-binary [4]. According to survey-based studies of children and adolescents, about 1.2–2.7% identify as transgender and 2.5–8.4% as TGD [2].

Despite a growing number of studies on transgender topics, a majority of this research still comes from the U.S. and Western Europe [2, 5]. This special issue includes studies from South America [6] and the Middle East [7] where there are a paucity of studies. International data is helpful for expanding global applicability of treatment guidelines such as the Standards of Care released by the World Professional Association for Transgender Health [2].

Studies from this collection show transgender and gender diverse (TGD) youth face significant minority stressors and mental health concerns [8–10]. For example, Haywood et al., found that many TDG youth still face high levels of non-acceptance and bullying even after social transition [8]. Another study found that the experience of trans hostility is associated with an increase in gender dysphoria and poor peer relations in TGD youth [9]. And, in a systematic review, TGD adolescents with gender dysphoria experienced a high co-occurrence of psychosocial and psychological vulnerability, leading to greater risk for suicidal ideation and life-threatening behaviors [10].

Previous research demonstrates that mental health symptoms and gender dysphoria improve with access to gender affirmative care including social, surgical and hormonal interventions [11–16]. In one study of nonbinary and gender diverse youth, use of puberty blockers and gender-affirming hormones were associated with 73% lower odds of suicidality and 60% lower odds of moderate to severe depression [13]. Importantly, access to gender-affirming hormones during adolescence has been found to improve mental health outcomes in adulthood [14]. And, surgical interventions such as chest reconstruction have been shown to improve dysphoria and body satisfaction in gender diverse youth [12, 16].

Yet, gender affirming care for youth is under attack. Legislative efforts to restrict access to gender affirming care are rampant, coordinated, and pervasive. Within the U.S. alone, nearly 39% of transgender youth live in states where there are bans on gender affirming care [17]. In the United Kingdom, life-saving treatments such as puberty blockers have been banned [18].

While credible scientific evidence about the positive benefits of gender affirming treatments struggle to find wide public dissemination [11–16], biased and methodologically flawed reports like the Cass Review are elevated within the public domain [19, 20]. Providers should understand, practice, and disseminate best practice guidance for the care of TGD youth as outlined by the American Endocrine Society [21] and the World Professional Association for Transgender Health [2].

Providers who care for TGD youth should strive to adopt inclusive models that value self-determination and an affirmative approach [22]. Defensive models of care that focus on fringe concerns such as transition regret and mitigatable side effects are not supported by international treatment guidelines [2]. Similarly, intrusive and/or prolonged assessments that interrogate a youth’s gender identity and delay social or medical transitions are more likely to cause harm than good [23]. Providers should strive to support transgender youth at all stages of their social, medical, and legal transitions, while empowering and supporting them toward authentic gender identity and expression.

## Data Availability

N/A.
